# DIDS Prevents Ischemic Membrane Degradation in Cultured Hippocampal Neurons by Inhibiting Matrix Metalloproteinase Release

**DOI:** 10.1371/journal.pone.0043995

**Published:** 2012-08-24

**Authors:** Matthew E. Pamenter, Julie Ryu, Serena T. Hua, Guy A. Perkins, Vincent L. Mendiola, Xiang Q. Gu, Mark H. Ellisman, Gabriel G. Haddad

**Affiliations:** 1 Division of Respiratory Medicine, Department of Pediatrics, University of California San Diego, La Jolla, California, United States of America; 2 National Center for Microscopy and Imaging Research, University of California San Diego, La Jolla, California, United States of America; 3 Center for Research in Biological Systems, University of California San Diego, La Jolla, California, United States of America; 4 Department of Neurosciences, University of California San Diego, La Jolla, California, United States of America; 5 The Rady Children's Hospital-San Diego, San Diego, California, United States of America; National Center for Scientific Research Demokritos, Greece

## Abstract

During stroke, cells in the infarct core exhibit rapid failure of their permeability barriers, which releases ions and inflammatory molecules that are deleterious to nearby tissue (the penumbra). Plasma membrane degradation is key to penumbral spread and is mediated by matrix metalloproteinases (MMPs), which are released via vesicular exocytosis into the extracellular fluid in response to stress. DIDS (4,4′-diisothiocyanatostilbene-2,2′-disulphonic acid) preserves membrane integrity in neurons challenged with an *in vitro* ischemic penumbral mimic (ischemic solution: IS) and we asked whether this action was mediated via inhibition of MMP activity. In cultured murine hippocampal neurons challenged with IS, intracellular proMMP-2 and -9 expression increased 4–10 fold and extracellular latent and active MMP isoform expression increased 2–22 fold. MMP-mediated extracellular gelatinolytic activity increased ∼20–50 fold, causing detachment of 32.1±4.5% of cells from the matrix and extensive plasma membrane degradation (>60% of cells took up vital dyes and >60% of plasma membranes were fragmented or blebbed). DIDS abolished cellular detachment and membrane degradation in neurons and the pathology-induced extracellular expression of latent and active MMPs. DIDS similarly inhibited extracellular MMP expression and cellular detachment induced by the pro-apoptotic agent staurosporine or the general proteinase agonist 4-aminophenylmercuric acetate (APMA). Conversely, DIDS-treatment did not impair stress-induced intracellular proMMP production, nor the intracellular cleavage of proMMP-2 to the active form, suggesting DIDS interferes with the vesicular extrusion of MMPs rather than directly inhibiting proteinase expression or activation. In support of this hypothesis, an antagonist of the V-type vesicular ATPase also inhibited extracellular MMP expression to a similar degree as DIDS. In addition, in a proteinase-independent model of vesicular exocytosis, DIDS prevented stimulus-evoked release of von Willebrand Factor from human umbilical vein endothelial cells. We conclude that DIDS inhibits MMP exocytosis and through this mechanism preserves neuronal membrane integrity during pathological stress.

## Introduction

Cells in the infarct core die within minutes of stroke onset, whereas in the surrounding region (the penumbra) death spreads slowly for hours to days post-insult [1], [2]. Unlike in the infarct core, the relatively slow propagation of cell death in the penumbra makes this region an attractive target for clinical rescue, particularly as the majority of stroke-related morbidity and mortality is attributable to progressive expansion of the infarct core [3]. The mechanisms of cell death in this region are poorly understood, but are likely initiated by deleterious alterations of the local perfusate following the release of cytoplasmic contents from ruptured core cells [4]. Indeed, loss of membrane integrity is a commonly-shared hallmark of cell-death pathways [5] and membrane cleavage facilitates the release of pro-apoptotic and -immunogenic signals, ions, and other debris from dying cells, which then accumulate in the local perfusate and initiate inflammatory and/or cell death pathways in adjacent cells [4], [6], [7]. In ischemic pathology, these effects are compounded by reduced cerebral blood flow following stroke, which limits O_2_ and nutrient delivery [1], and slows the removal of extruded signaling molecules, ions, and metabolically-derived lactate and CO_2_; thereby enhancing cytotoxicity, ionic imbalance, and acute acidification in the penumbral milieu [7], [8], [9]. Thus, penumbral cells are exquisitely vulnerable to pro-apoptotic or -inflammatory signals released from ruptured cells in the nearby infarct core; and the mechanisms underlying cell rupture likely play an important role in the spread of cell death and inflammation following stroke.

Matrix metalloproteinases (MMPs) are a family of >20 zinc-dependent enzymes that cleave most components of the extracellular matrix and regulate matrix remodeling during normal CNS development and repair [10]. MMPs also play key roles in physiological and pathophysiological processes involved in neuroinflammation and ischemia, endotoxin shock, multiple sclerosis, bacterial meningitis, wound healing, bone remodeling, organogenesis, and cancer cell invasion, among others [11], [12], [13], [14]. MMPs are synthesized in the ER as inactive preproenzymes and are converted to inactive proenzymes during translation. Most proMMPs are stored in this latent form in the cytosol, bound to their specific inhibitors (tissue inhibitor of metalloproteinases: TIMPs) [15]. In response to cellular signals, many MMPs become dissociated from TIMPs (e.g. MMP-9) and are secreted into the extracellular fluid (ECF) as inactive pro-enzymes via vesicular exocytosis, where they become activated by proteolytic digestion of a short-chain amino-terminal pro-peptide by other proteases, and then act extracellularly to cleave plasma membranes, detach cells from the matrix, and induce further MMP production [10]. In addition, other members of the MMP family are activated in the cytosol (e.g. MMP-2), while an additional family of MMPs are membrane bound proteins (MT-MMPs) that contribute to the activation of proMMP-2 [15]. Through these actions MMPs underlie the dismantling and removal of damaged cells following pathological insults [10], [12], [13]. In addition to cleaving the extracellular matrix, MMPs also process a variety of bioactive molecules, including the pro-forms of other MMPs and of immunogenic molecules such as cytokines (e.g. interleukin-1β (IL-1β); tumor necrosis factor-α) and neurotrophins (e.g. nerve growth factor); and through these actions MMPs also play a key role in the initiation and regulation of inflammatory pathways critical to infarct expansion into the penumbra [10], [12], [16].

During ischemic pathology in brain the expression and activity of the gelatinases MMP-2 (gelatinase A) and -9 (gelatinase B) in particular are elevated, and contribute to blood-brain barrier disruption, microvascular matrix and also permeability barrier (plasma membrane) degradation, and activation of neuroinflamatory pathways [17], [18], [19], [20], [21], [22]. Interestingly, the kinetics of MMP activation and spreading cell death following stroke are similar: in the infarct core gelatinases are activated within minutes of middle cerebral artery occlusion (MCAO), whereas in the penumbra, their activation is delayed by hours and follows the spread of cell death [23]. Conversely, gelatinase inhibitors or MMP-9^−/−^ are neuroprotective against MCAO in mice and also reduce IL-1β-mediated systemic neuroinflammation [16], [24], [25], [26], [27]. The cytoprotective mechanisms of MMP inhibition have not been elucidated; however, inhibition of MMP-mediated cleavage of plasma membrane and pro-enzymes would reduce the secretion and activation of pro-apoptotic and immunogenic signals into the penumbral milieu, and retard the spread of cell death and neuroinflammation following ischemic insult.

Recently our laboratory reported that the anion channel antagonist 4,4′-diisothiocyanatostilbene-2,2′-disulphonic acid (DIDS, 400 µM) preserves neuronal membrane integrity and prevents propidium iodide (PI) uptake and lactate dehydrogenase release, and also IL-1β mRNA expression, from primary mouse hippocampal and cortical neuronal cultures and cell lines challenged with either 24-hrs of a novel ischemic penumbral mimic (ischemic solution: IS [7]), or a 5-day hypoxic insult (1% O_2_) [28], [29], [30]. Also, during routine culture maintenance, we observed that DIDS-treated cells took longer to detach from the growth matrix when treated with the proteinase trypsin. Based on these observations we hypothesized that DIDS impairs proteinase activity, and that through this action, prevents membrane degradation characteristic of ischemic pathology. To examine this hypothesis we observed the effects of IS or staurosporine (STS) treatment±DIDS (0.01–4.0 mM) on cellular detachment, plasma membrane integrity, and MMP-2 and -9 protein expression in murine hippocampal neuronal cultures. We also examined the ability of DIDS to affect the activity of the potent general proteinase agonist 4-aminophenylmercuric acetate (APMA) and examined this question in several commonly studied cell lines to determine whether the putative inhibitory effect of DIDS on proteinase activity is ubiquitous between cell types. Finally, since MMPs are released via vesicular exocytosis, we tested the ability of DIDS to interfere with the vesicular release of von Willebrand Factor (vWF) from human umbilical vein endothelial cells (HUVECs), a well-understood physiologically relevant model of non-pathological vesicular exocytosis that does not involve proteinase activity [31].

## Materials and Methods

### Cell cultures

HT22 mouse hippocampal neurons (a gift from Dr. Pam Maher, Salk Institute, La Jolla, CA, [32]) and C8D1A mouse type-I astrocytes (ATCC, Manassas, VA) were cultured in Dulbecco's Modified Eagle Medium (DMEM, ATCC) supplemented with 10% bovine calf serum (Hyclone, Santa Clara, CA) and 100 U/ml penicillin/streptomycin (Invitrogen, Carlsbad, CA) and grown at 37°C in a 5% CO_2_ incubator. Human embryonic kidney (HEK 293, ATCC), mouse embryonic mesenchymal (C3H-10T1/2, a gift from Dr. He Huang, UCSD, La Jolla, CA, [33]), mouse type-I astrocyte clones (C8D1A, ATCC), and HeLa cells (ATCC) were grown in the same conditions. Human umbilical vein endothelial cells (HUVECs, ATCC) were grown in Endothelial Basal Medium (EBM) supplemented with Endothelial Growth Medium (EGM-2-MV) BulletKit (Lonza, Walkersville, MD). PC12 cells (ATCC) were grown in 75 cm^2^ flasks coated with collagen I (Greiner Bio-One, Monroe, NC) and fed F12K culture medium (ATCC) supplemented with 15% horse serum (ATCC), 2.5% fetal bovine serum, and 0.5% penicillin G (Invitrogen). PC12 cells were treated for 24–48 hrs prior to experimentation with F12K medium with 0.5% penicillin G/streptomycin (pen/strep), 5 ng/ml nerve growth factor, and 1% horse serum to induce differentiation into a neuronal phenotype. All cells were grown for 5–8 passages and split when they reached 60–80% confluence. For experiments, cells were seeded into 96-well microplates (Corning, Lowell, MA), glass-bottom 12-well microplates or 35 mm culture dishes (MatTek, Ashland, MA), or cell culture flasks at a density such that when grown overnight they reached ∼70% confluence for experimentation. Samples were treated as specified in the *experimental design* section (below). To reduce sheer stress, cells seeded into 96-well microplates were gently washed with a TECAN PW96/384 Washer (TECAN, San Jose, CA) and then examined visually to ensure cells had not been washed away.

### Experimental design

Samples were treated with DIDS (400 µM, unless otherwise indicated) for 6 or 24 hrs (as indicated) in three primary treatment categories: (1) cell death-negative control (DMEM/F12 media (Invitrogen) supplemented with 2% fetal bovine serum and 1% pen/strep, gassed with 21% O_2_, 5% CO_2_, balance N_2_), (2) an ischemic penumbral perfusate mimic (IS, in mM: K^+^ 64, Na^+^ 51, Cl^−^ 77.5, Ca^2+^ 0.13, Mg^2+^ 1.5, glucose 3.0, glutamate 0.1, [315 mOsM, pH 6.5, 1.5% O_2_, 15% CO_2_, balance N_2_]), or (3) cell death-positive control (DMEM/F12 containing the pro-apoptotic agent STS (2.5 µM)). DIDS-free controls were conducted in parallel for each experimental paradigm and all treatments were run simultaneously for each assay. 400 µM DIDS was utilized in most experiments as this concentration has previously been shown to be effective at preserving plasma membrane viability against IS and hypoxic insults in primary hippocampal and cortical cultures [28], [29], [30]. Since MMP activity is involved in normal cellular maintenance and division, we pre-treated samples for 6-hrs with serum-free media prior to treatment onset in order to isolate pathology-mediated changes in MMP expression. In some experiments, cells were stimulated with the general protease agonist 4-aminophenylmercuric acetate (APMA, 100 µM) or the vesicular (V-type) H-ATPase inhibitor bafilomycin A (BMA, 100 nM). HUVEC vWF release was stimulated with the Ca^2+^ ionophore A23187 (10 µM) and vWF formation was inhibited with brefeldin A (BFA, 1 µg/ml), which interferes with protein trafficking from ER to Golgi. In APMA-treated experiments, samples were not pre-treated with serum-free media prior to treatment onset because a) this treatment did not involve pathology-induced MMP activity, and b) the cellular response to APMA was highly robust and MMP expression changes were easily detected. Following treatment, samples were assayed as indicated below. A23187, APMA, BFA, BMA, DIDS, and STS were dissolved in DMSO to a final bath [DMSO]<0.01%, and all solutions were made fresh daily. Chemicals were purchased from Sigma unless otherwise indicated (Sigma-Aldrich, St. Louis, MO).

### Adenylate kinase membrane viability assay

Assessment of the leakage of bulky adenylate kinase (AK) through plasma membranes was measured using a 96-well Toxilight microplate kit according to the manufacturer's instructions (Lonza). Briefly, cells were grown and subsequently treated in 96-well microplates and total free AK was assessed via a luciferase assay in each well before and after cell lysis buffer addition and homogenization, to determine released and total cellular AK, respectively. AK release is expressed relative to total AK in each well.

### Cellular detachment assay

Samples were grown in 150 cm^2^ cell culture flasks and following 24-hrs treatment supernatant aliquots were taken to assay detached cell density in the ECF. The remaining treatment media was aspirated and adherent cells were rinsed in PBS and then detached from the matrix by 7–10 mins incubation at 37°C in trypLE express (Gibco, for HUVECs), 0.05% trypsin with EDTA (Invitrogen, for neurons), or 0.25% trypsin with EDTA (Invitrogen) for all other cell lines. Cells were re-suspended in 4 volumes of serum-free DMEM and centrifuged for 5 mins at 200 x *g*. The resulting supernatant was discarded and the cell pellet re-suspended in PBS. Treatment supernatant samples or cell suspension aliquots were gently mixed and then counted immediately on a hemocytometer. Cells were counted in 5 fields from each side of the hemocytometer for each sample and 3 samples were assayed per treatment paradigm. The total number of cells in each sample was assessed as cells_attached_+cells_ECF_, and percent cellular detachment was determined. Compared to their respective controls, DIDS-treated samples were desensitized to trypsin in each treatment paradigm, requiring longer incubation times to induce detachment. To ensure that this longer incubation did not induce significant cell death and confound our results, we incubated control cells in trypsin for 10 or 30 mins in separate control experiments. Cell viability decreased ∼5% between 10- and 30-min treated samples; however, this error is small compared to the large changes we observed between treatment groups, and thus we do not consider it to be significant source of error in our experiments. Experiments were repeated 8–10 times for each treatment group.

### Confocal microscopy

Fixed samples were imaged on an Olympus FV1000 scanning confocal microscope, using 572 nm (TRITC), 488 nm (FITC), and 405 nm (DAPI) laser lines (Olympus, San Diego, CA). For data collection the parameters of the microscope such as light intensity, exposure time, camera gain, etc., were determined for the brightest fluorescing sample and standardized for subsequent samples. For co-localization analysis five random sections from each study group were taken at 10x magnification using AxioVision (Carl Zeiss, Thornwood, NY), and the percentage of neurons staining positive for MMP-2 and -9 or PI uptake was determined by the ratio of FITC-stained cells to DAPI-stained nuclei. Metamorph (Molecular Devices, Sunnyvale, CA) image analysis software was used to count fluorophore-positive stained cells/DAPI-positive cells. For vWF releases images Z-projections from 8 optical sections taken 4 µm apart were created by averaging pixel intensity at each pixel position using Image J (NIH).

### ELISA

HUVEC vWF release was quantified using a human vWF ELISA kit according to the manufacturer's protocol (Sino Biological Inc., Beijing, CH), using mouse anti-vWF monoclonal antibody and biotinylated rabbit anti-vWF polyclonal antibody as the capture and detection antibodies, respectively. Samples were assayed in triplicate and ELISA analysis was repeated 2 times for each experiment and treatment.

### Immunohistochemistry

Samples grown in glass-bottom 35-mm dishes were treated for 6-hrs as indicated and then fixed with 4% paraformaldehyde in PBS for 15 mins at room temperature. Samples were washed with PBS (3×5 mins) and then incubated in blocking buffer (10% normal goat serum and 0.3% Triton X-100 (Sigma)) for 30 mins. Samples were then incubated with 10 µg/ml mouse MMP-2 or MMP-9 polyclonal antibodies, goat IgG (R&D, Minneapolis, MN), diluted in ½ blocking buffer for 24 hrs at 4°C. Following incubation, samples were washed in PBS+0.1% Triton X-100 (3×10 mins) and then incubated with anti-goat Alexa Fluor 488-conjugated secondary antibody (Invitrogen), diluted 1∶100 in ½ blocking buffer for 1 hr at RT. Finally, samples were washed in PBS+0.1% Triton X-100 (3×10 mins), mounted with Prolong Vectashield (with DAPI, unless otherwise indicated, Invitrogen), cover-slipped and sealed with nail polish. Samples were stored in the dark at 4°C and imaged within one week. Experiments were repeated 3 times for each treatment.

### Protein extraction

Samples grown in 150 cm^2^ culture flasks were treated for 6-hrs and then rinsed twice with PBS and detached from the matrix with a cell scrapper into ice-cold PBS. The resulting cell suspensions were centrifuged at 250 x *g* for 5 mins at 4°C, the supernatant was aspirated away, and cells were re-suspended in cell lysis buffer. Samples were then homogenized by vortexing for 60 seconds and proteins were extracted by incubation in lysis buffer with mixing at 4°C for 45 mins, followed by centrifugation for 10 mins at 14,000 x *g* at 4°C. Supernatants were taken as whole cell lysates and protein concentration was measured using a bicinchoninic acid kit, according to the manufacturer's instructions (Sigma).

### Transmission electron microscopy

Samples in 35 mm #0 thickness culture dishes were fixed with a 37°C solution of 2% paraformaldehyde, 2.5% glutaraldehyde (Ted Pella, Redding, CA) in 0.1 M sodium cacodylate (pH 7.4), and transferred to room temperature for 10 mins, and then incubated for an additional 30 mins on ice. Fixed cultures were rinsed 3 times for 3 mins each with 0.1 M sodium cacodylate plus 3 mM CaCl_2_ (pH 7.4) on ice and then post-fixed with 1% osmium tetroxide (Ted Pella), 0.8% potassium ferrocyanide, and 3 mM CaCl_2_ in 0.1 M sodium cacodylate (pH 7.4) for 60 mins, and were then washed 3 times for 3 mins with ice-cold distilled water. Cultures were finally stained overnight with 2% uranyl acetate at 4°C, dehydrated in graded ethanol baths, and embedded in Durcupan resin (Fluka, St. Louis, MO). Ultrathin (70 nm) sections were post-stained with uranyl acetate and lead salts, and evaluated by a JEOL 1200FX transmission electron microscopy operated at 80 kV. Images were recorded on film at 6,000x magnification. The negatives were digitized at 1,800 dpi using a Nikon Cool scan system, giving an image size of 4033×6010 pixel array and a pixel resolution of 2.35 nm [34]. Images of 20 cell membranes were taken from each experimental condition. All TEM experiments were repeated twice. Plasma membrane thickness was measured at 10 randomly chosen locations from each of 5 images of cells chosen from each experimental replicate. Blebbing was quantified by normalizing the number of membrane blebs in each image to the total perimeter of the plasma membrane.

### Time-lapse confocal microscopy

Neurons were seeded into 12-well #1.5 thickness glass bottom microplates (MatTek). Cells were maintained at 37°C with either 21%O_2_/5% CO_2_ (normoxia) or 1.5% O_2_/15% CO_2_ (IS) for the duration of the experiment and were treated as indicated in the *Results* section. For each well, three regions were chosen at random and differential interference contrast (DIC) images were taken with 10× and 20× air objectives on a Perkin Elmer Ultraview Vox spinning disk confocal microscope (Perkin Elmer, Waltham, MA) at 5 min intervals for 24 hrs. Data were analyzed using Volocity software (Perkin Elmer). Experiments were repeated 3 times for each experimental condition.

### Vital dye exclusion membrane viability assays

Membrane viability was assessed following 24-hrs treatment as the ability of cells to exclude the vital dyes propidium iodide (PI) or trypan blue (TB). For confocal microscopy, samples were treated and then incubated in 5 ng/ml PI for 15 mins before being rinsed and fixed as described in the *Immunohistochemistry* section (above). PI exclusion was determined by the ratio of PI-positive stained cells to DAPI-stained nuclei and imaging experiments were repeated 3 times for each treatment. The dose-dependent response of DIDS on IS-induced PI uptake was assessed using a high-throughput 96-well microplate-based assay. PI uptake was assessed immediately following experimental treatment on a Bio-Tek PowerWave 340 microplate spectrophotometer (Bio-Tek, Winooski, VT, Ex/Em: 485/630 nm) and analyzed using Gen 5 software (Bio-Tek). Microplate PI experiments were repeated 10 times and each plate contained 16 replicate wells each of control-, IS-, or STS-treated samples±DIDS (0.04, 0.40, and 1.0 mM). Blank wells and cell-free wells containing each treatment perfusate with PI were also included on each plate and the final data is corrected for these factors. For TB exclusion analysis, treatment supernatant samples or cell suspension aliquots were obtained as detailed in the *cellular detachment* methods (above) and were gently mixed in an equal volume of 0.4% TB (Gibco) for 3 mins at room temperature, and then counted immediately on a hemocytometer. Unstained (viable) and stained (dead) cells were counted in 5 fields from each side of the hemocytometer for each sample and 3 samples were assayed per treatment paradigm. Cells were counted from 3–5 flasks for each treatment.

### Western blots

Equal amounts of protein (40 µg/well) were separated on 4–12% precast NuPAGE bis-Tris SDS-PAGE gels (Invitrogen) and transferred to polyvinylidene difluoride membranes (Immobilin-P; Millipore, Bedford, MA). Western blots were performed with antibodies against α-actin (1∶2,000, Cell Signaling, Danvers, MA); MMP-2 and MMP-9 (1∶500, Cell Signaling); and vWF (1∶500, DakoCytomation, Carpinteria, CA). Specific bands were visualized after incubation with the respective secondary antibodies using enhanced chemiluminescense (GE Healthcare/Amersham Biosciences, Buckinghamshire, UK). Densitometry of Western blots from each experimental group were obtained (*n* = 3–5 for each), and absolute values were normalized to α-actin for cell-derived protein samples. Results were analyzed in arbitrary units, comparing each value with that obtained from each respective α-actin measurement on each blot. Supernatant protein samples did not contain α-actin and were normalized to total protein loaded in each well. Results are expressed as fold-change relative to normoxic controls run simultaneously.

### Zymography

Substrate specific zymography for determination of gelatinolytic activity of MMP-2 and MMP-9 was performed under denaturing but non-reducing conditions as follows. Samples of cell fractions (isolated as described for Western blot analysis above) or ECF from various cell lines were mixed with 2× loading buffer (400 mM Tris-HCl, pH 6.8, 5% SDS, 20% Glycerol, 0.006% bromophenol blue) and 40 µl aliquots were applied onto a 10% polyacrylamide gel containing 0.1% gelatin. Electrophoresis was performed at 25-mA constant current for 2 hrs at room temperature, followed by a 1 hr equilibration of the gels in 2.5% TX-100 to remove SDS. The gels were incubated in enzyme buffer (50 mM Tris HCl, pH 7.3, 200 mM NaCl, 5 mM CaCl_2_, and 0.02% Brij 35) for 48 hrs at 37°C to activate gelatinolytic activity of MMPs. Enzymatic bands were visualized by negative staining of the gel with an aqueous solution of 0.5% Coomassie brilliant blue dye (in: 50% methanol, 10% acetic acid, and 40% de-ionized water). Gels were destained for 2×20 mins in a mixture consisting of 20% methanol, 10% acetic acid, and 70% de-ionized water. Molecular sizes of the bands displaying enzymatic activity were identified by comparison to pre-stained standard proteins (New England BioLabs, Ipswich, MA) and densitometry was performed using Biorad imaging software (Biorad, Hercules, CA). Experiments were repeated 4 times.

### Statistics

Data were analyzed using a two-tailed Student *t*-test or one-way analysis of variance (ANOVA), followed by Dunnet's post-test. Significances were indicated if *P*<0.05 assuming two groups had an equal variance. Statistical analysis was performed using Prism software (GraphPad, San Diego, CA).

## Results

### DIDS reduces pathology-induced plasma membrane degradation in neurons

**Figure 1:**
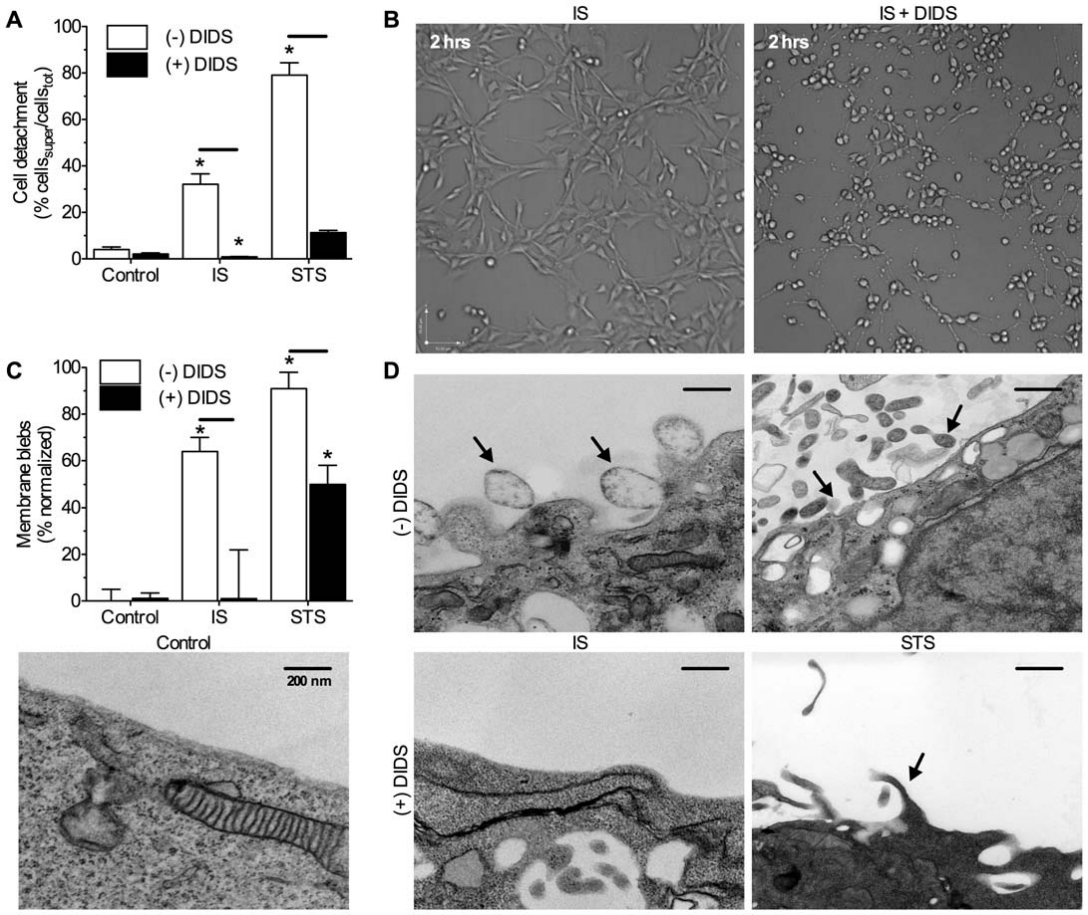
DIDS ameliorates pathology-induced neuronal detachment and plasma membrane blebbing. (**A**) Summary of the effect of DIDS on stress-induced cellular detachment from the matrix expressed as the percentage of total neurons in each experiment that detached to the supernatant following 24-hrs. (**B**) 10x DIC images from 24-hr time-lapse recordings of neurons following 2-hrs of treatment as indicated (see also 24-hr supplementary videos online). (**C**) Summary of the effect of DIDS on stress-induced plasma membrane blebbing expressed as the perimeter of membrane blebs relative to the total perimeter of the plasma membrane in each image. (**D**) Sample TEM images of plasma membranes from (C). TEM images are oriented with the cell interior at the bottom of the image. Arrows indicate blebbing events. Data are mean ±SEM from 8–10 separate 24-hr experiments. Asterisks (*) indicate significant difference from normoxic controls; black bars indicate significance between connected treatments (*p*<0.05). Treatments: ischemic solution (IS), 2.5 µm staurosporine (STS), and 400 µm DIDS.

Through 24-hrs, control cells retained typical neuronal phenotypes, including the maintenance of synaptic processes, and cellular attachment to the growth matrix (*n* = 8–10 for all treatment conditions, Fig. 1A&B, Videos S1, S2, S3). Conversely, IS-challenged cells rounded off and appeared unhealthy, and 32.1±4.5% of cells detached from the matrix at 24-hrs. Neurons treated with the pro-apoptotic agent STS rapidly rounded off and 79.1±5.3% were detached at 24-hrs. DIDS-treated (400 µM) cells rounded off and exhibited extensive retraction of processes within 2-hrs of treatment (Fig. 1B, and see also 24-hr time-lapse Videos S1, S2, S3); however, these cells did not detach from the matrix or exhibit membrane blebs (Fig 1A&C). Similarly, DIDS almost entirely abolished IS- or STS-mediated cellular detachment in all experiments (Fig. 1A), and also ameliorated pathology-induced plasma membrane degradation (Fig. 1C&D). Relative to controls, IS or STS-challenged neurons exhibited extensive blebbing of the plasma membrane (>60% of plasma membrane surface area ruptured, *n* = 10 for each treatment condition, Fig. 1C&D arrows), while DIDS entirely abolished IS-induced membrane blebbing and reduced STS-mediated blebbing ∼50%.

### DIDS abolishes pathology-induced vital dye uptake

**Figure 2:**
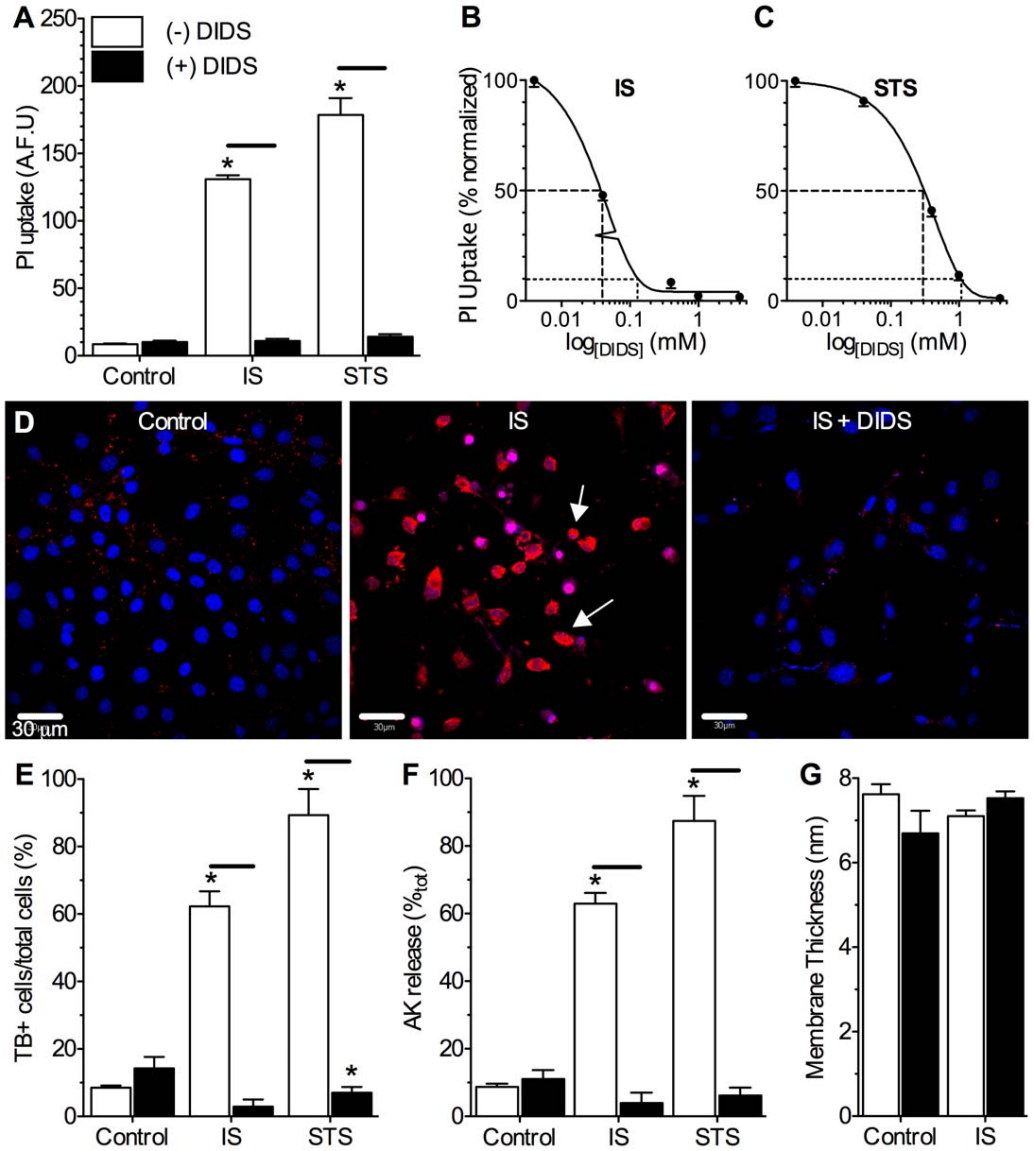
DIDS abolishes pathology-induced membrane degradation. Stress-induced vital dye uptake and adenylate kinase release are prevented by DIDS treatment. (**A**) Summary of the effect of DIDS on stress-induced propidium iodide (PI) uptake. (**B&C**) Dose-response relationship of IS- (B) or STS-mediated (C) PI uptake vs. [DIDS]. (**D**) Confocal fluorescence images of PI fluorescence (red) from neurons treated as indicated. Nuclei were stained with DAPI (blue) for co-localization analysis. Arrows indicate representative neurons that have taken up PI. (**E**) Summary of the effect of DIDS on stress-induced trypan blue (TB) uptake. (**F**) Summary of the effect of DIDS on stress-induced adenylate kinase (AK) release. (**G**) Summary of plasma membrane widths measured from TEM analysis. Data are mean ±SEM from 3-10 separate 24-hr experiments. Asterisks (*) indicate significant difference from normoxic controls; black bars indicate significance between connected treatments (*p*<0.05). Treatments as per Fig. 1 caption except where indicated otherwise.

To confirm that the effect of DIDS on pathology-induced blebbing correlated with preservation of membrane integrity as a permeability barrier, we examined the ability of cells to exclude vital dyes and retain adenylate kinase (AK). Control cells or cells treated with DIDS alone excluded propidium iodide (PI, *n* = 4–6 for each treatment, Fig. 2A–D) and trypan blue (TB, *n* = 3–5 for each treatment, Fig. 2E), and did not release AK (*n* = 3 for each treatment, Fig. 2F); whereas pathology-challenged cells took up PI and TB, and IS- or STS-treatment caused ∼60–90% of total cellular AK to be released from neurons at 24-hrs. Co-treatment with DIDS prevented pathology-mediated vital dye uptake in a dose-dependent manner, and abolished AK release during IS- or STS-treatment. None of the treatments examined had a significant effect on plasma membrane widths (*n* = 10 for each, Fig. 2G). Since pathology-induced cellular detachment and plasma membrane cleavage are both primarily mediated by MMP activity [15], [25], we next examined whether DIDS prevented the activation or impaired the function of these enzymes.

### Pathological insults increase intracellular neuronal MMP protein expression

**Figure 3:**
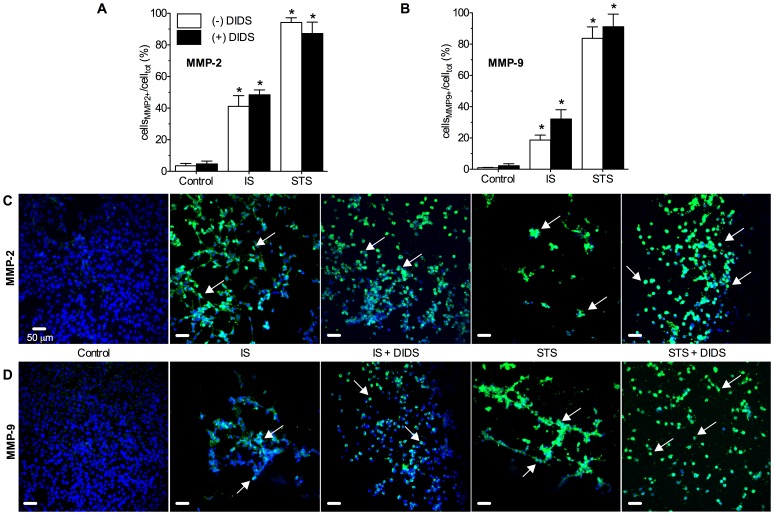
DIDS does not prevent pathology-induced neuronal MMP-2 or -9 protein expression. IS or STS treatment increase neuronal MMP-2 and -9 protein expression, and these changes are unaffected by co-treatment with DIDS. (**A&B**) Summaries of immunohistochemical (IHC) analysis, and (**C&D**), sample confocal IHC fluorescent images of neurons stained positive for MMP-2 (A&C) and MMP-9 (B&D) (green fluorescence). Nuclei were stained with DAPI (blue) for co-localization analysis. Arrows indicate representative MMP+ staining. Summary data is presented as the percentage of MMP+ cells relative to the total number of neurons in each experiment (as determined by DAPI fluorescence). Data are mean ±SEM from 3–5 separate 6-hr experiments. Asterisks (*) indicate significant difference from normoxic controls (*p*<0.05). Treatments as per Fig. 1 caption.

To assess the effect of pathological treatments and DIDS on cellular proteinase activity, we examined stress-induced changes in neuronal expression of MMP-2 and -9 proteins. At 24-hrs, pathologically-challenged neurons were too degraded to extract high quality protein for molecular analysis; and so in other experiments we assayed neurons following 6-hrs treatment to provide insight into changes that contribute to the observed cell death phenotypes at 24-hrs. For these experiments, samples were perfused with serum-free media for 6-hrs prior to treatment onset in order to arrest cell division and minimize maintenance-related MMP activity in samples. In this manner pathology-induced MMP activity could be determined. In immunohistochemical (IHC) analysis of cells expressing MMPs the prevalence of neurons stained positive for MMP-2 and -9 was increased relative to un-treated controls following 6-hours of IS- or STS-treatment (*n* = 3 each, Fig. 3A–D). Co-treatment with DIDS had no effect on the pathology-induced increase in MMP-positive cells, and DIDS did not effect either MMP-2 or -9 staining in control, IS or STS treated neurons.

**Figure 4:**
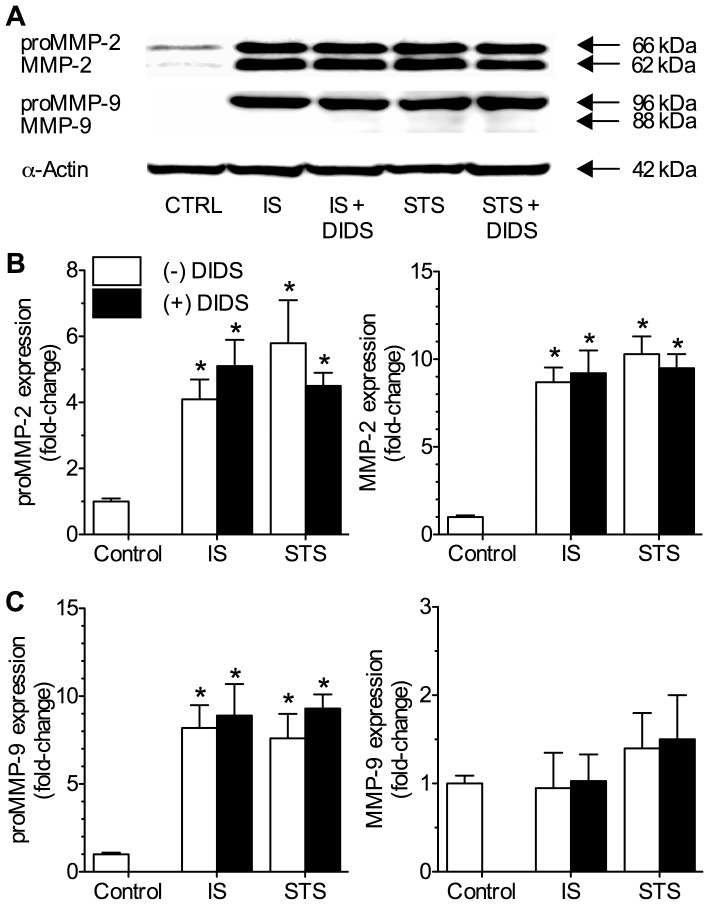
DIDS does not effect intracellular expression of pro- or active MMP-2 or -9 isoforms. IS and STS treatment increased intracellular expression of proMMP-2 and -9 isoforms, as well as the active form of MMP-2. DIDS did not effect these changes. (**A**) Sample Western blots of MMP-2 and -9 protein expression. Latent and active isoforms of MMPs were detected at 66 and 62 kDA (MMP-2) and 96 and 88 kDA (MMP-9), respectively. (**B&C**) Summaries of neuronal latent and active MMP-2 (B) and MMP-9 (C) protein isoform expression from analysis of cellular fractions normalized to the cellular expression of α-actin on the same blot. Data are presented as fold-change relative to untreated controls. Data are mean ±SEM from 3-5 separate 6-hr experiments. Asterisks (*) indicate significant difference from normoxic controls (*p*<0.05). Treatments as per Fig. 1 caption.

These results were confirmed with Western blot analysis of identically treated neuronal populations. In these experiments intracellular proMMP-2 protein isoform expression was increased 4 to 6-fold relative to untreated controls following 6-hours of either IS or STS-treatment, while expression of the active isoform of MMP-2 increased 8 to 10-fold in the same samples (*n* = 4 each, Fig. 4A-B). Conversely, IS or STS treatment increased intracellular proMMP-9 expression 7 to 10-fold at the same time point but did not increase expression of the active isoform of MMP-9 (Fig. 4A&C), which was expected since proMMP-9 is activated extracellularly. Co-treatment with DIDS during either IS- or STS-treatment had no effect on the pathology-mediated increases in intracellular latent and active MMP-2 and proMMP-9 expression.

### DIDS prevents pathology-mediated expression of MMP proteins in the ECF

**Figure 5:**
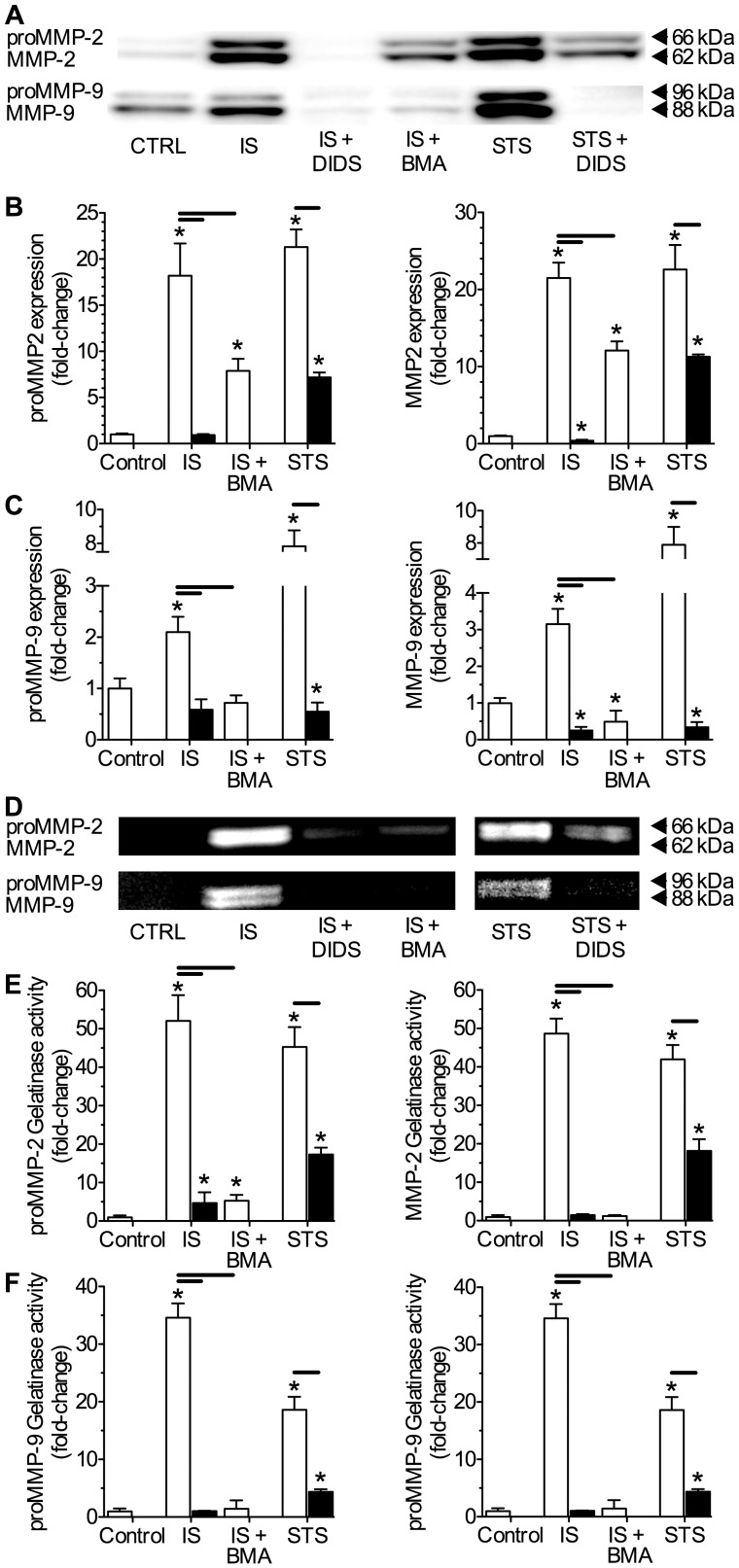
DIDS or V-ATPase inhibition reduce or abolish stress-mediated extracellular MMP-2 or -9 protein expression and gelatinolytic activity. IS or STS treatment increase the extracellular accumulation and gelatinolytic activity of latent and active MMP-2 and -9 proteins. DIDS or the specific vesicular ATPase (V-ATPase) antagonist bafilomycin A (BMA) each prevent or reduce extracellular latent and active MMP-2 and -9 isoform expression and gelatinolytic activity. (**A**) Sample Western blots of MMP-2 and -9 protein expression, and (**B&C**) summaries of neuronal latent and active MMP-2 (B) and MMP-9 (C) protein expression from analysis of supernatant fractions normalized to untreated controls. (**D**) Sample zymography gel of MMP-2 and MMP-9 gelatinolytic activity of supernatant samples taken from neurons treated as indicated. (**E&F**) Summaries of latent and active MMP-2 (E) and -9 (F) isoform gelatinolytic activities from (D). Latent and active forms of MMPs were detected in these assays at 66 and 62 kDA (MMP-2) and 96 and 88 kDA (MMP-9), respectively. Data are presented as fold-change relative to untreated controls. Data are mean ±SEM from 4 separate 6-hr experiments. Asterisks (*) indicate significant difference from normoxic controls; black bars indicate significance between connected treatments (*p*<0.05). Treatments as per Fig. 1 caption and 100 nM BMA.

Since DIDS did not interfere with stress-mediated intracellular MMP protein expression, we next quantified MMP-2 and -9 proteins secreted into the ECF. In ECF perfusate samples collected from cells challenged for 6-hrs with IS or STS, latent and active MMP-2 and -9 protein isoform expression was increased in Western blot analysis, and DIDS significantly reduced or entirely abolished IS- or STS-induced MMP expression (*n* = 4, Fig. 5A–C). Relative to controls, the extracellular expression of MMP-2 proteins was more strongly upregulated by stress treatments than that of MMP-9, and both latent and active MMP-2 isoforms increased ∼18 to 22-fold depending on the stress applied, whereas latent and active MMP-9 increased ∼2 to 8-fold relative to untreated controls. Interestingly, some expression of the latent and active forms of MMP-9 was detected in control ECF samples (Fig. 5A), which may be related to cellular maintenance or stress due to the growth-arresting serum starvation pretreatment paradigm applied to these samples.

DIDS had a similar effect on MMP gelatinolytic activity in supernatant samples collected from pathology-challenged neurons. Here, gelatinolytic activity was increased ∼20 to 50-fold by IS- or STS-treatment at bands corresponding to the latent and active forms of MMP-2 and MMP-9 (*n* = 4, Fig. 5D-F), consistent with increased extracellular expression of both MMP isoforms in stress-treated cells (Fig. 5A–C). In IS- and STS-treated samples co-treated with DIDS, gelatinase activity of both latent and active MMP-2 and -9 isoforms was markedly reduced or entirely abolished. The vesicular V-type ATPase antagonist bafilomycin A (BMA) similarly reduced IS-mediated latent and active MMP-2 and -9 protein expression in the ECF and abolished related supernatant gelatinase activity (Fig. 5A–F), suggesting a central role for vesicular release in ischemic MMP efflux and activation.

### DIDS reduces APMA-induced MMP protein expression and gelatinolytic activity in the extracellular fluid

**Figure 6:**
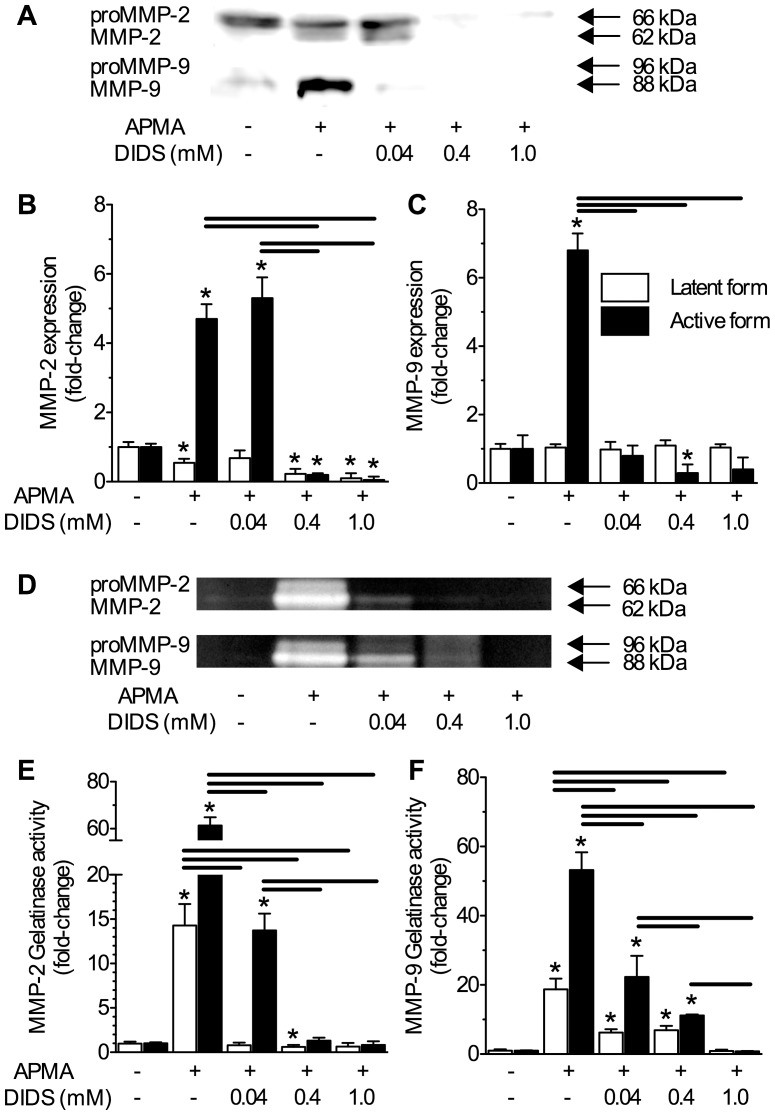
DIDS inhibits APMA-mediated extracellular MMP-2 or -9 protein expression and gelatinolytic activity in a dose-dependent fashion. Perfusion of the general proteinase agonist 4-aminophenylmercuric acetate (APMA) increases latent and active MMP-2 and active MMP-9 isoform expression and extracellular gelatinolytic activity in the supernatant. Co-treatment with DIDS reduces extracellular MMP protein expression and gelatinolytic activity in a dose-dependent fashion. (**A**) Sample Western blot of MMP-2 and -9 protein expression, and (**B&C**) summaries of dose-response relationships of APMA-mediated latent and active MMP-2 (B) and -9 (C) protein isoform expression in supernatant fractions vs. [DIDS] (0.04–1.0 mM), normalized to untreated controls. (**D**) Sample zymography gel of APMA-mediated latent (white bars) and active (black bars) MMP-2 and -9 isoform gelatinolytic activity vs. [DIDS] of supernatant samples taken from neurons treated as indicated. (**E&F**) Summaries of latent and active MMP-2 (E) and -9 (F) isoform gelatinolytic activities from (D). Data are presented as mean fold-change relative to untreated controls. Data are mean ±SEM from 3–4 separate 1-hr experiments. Asterisks (*) indicate significant difference from normoxic controls; black bars indicate significance between connected treatments (*p*<0.05). Treatments as per Fig. 1 caption and 100 µM APMA.

Since pathology-mediated cytotoxicity involves myriads of pathways and interactions in addition to MMPs, we next examined the efficacy and dose-dependency of DIDS as an inhibitor of MMP activation by co-treating normoxic neurons with the potent general proteinase agonist 4-aminophenylmercuric acetate (APMA, 100 µM). Cells were treated with DMEM±APMA ±DIDS (0.04, 0.4, or 1.0 mM) and the findings from these experiments were consistent with those from the pathological experiments above. One hour of APMA-treatment induced marked increases of active MMP-2 and MMP-9 protein expression in Western blot analysis of ECF samples (*n* = 3, Fig. 6A–C); and at moderate (0.4 mM) or high (1.0 mM) concentrations, DIDS nearly abolished APMA-mediated extracellular expression of active MMP-2 and -9 isoforms. Interestingly, extracellular expression of proMMP-2 decreased progressively with APMA treatment, and proMMP-9 was not detected in these samples. These observations are likely due to rapid activation of latent MMP isoforms by APMA. Similar to Western blot analysis, APMA induced gelatinolytic activity in the ECF, and in all experiments, DIDS negatively regulated this gelatinolytic activity in a dose-dependent fashion (*n* = 4, Fig. 6D–F). Notably, APMA-induced MMP activity was highly robust and these experiments did not involve pathology-mediated activation of MMP-related pathways; therefore, Western blot samples were not pre-treated with serum-free media. As a result, control-treated neurons exhibited mild to moderate proMMP-2 expression, likely due to ongoing cellular maintenance processes.

**Figure 7:**
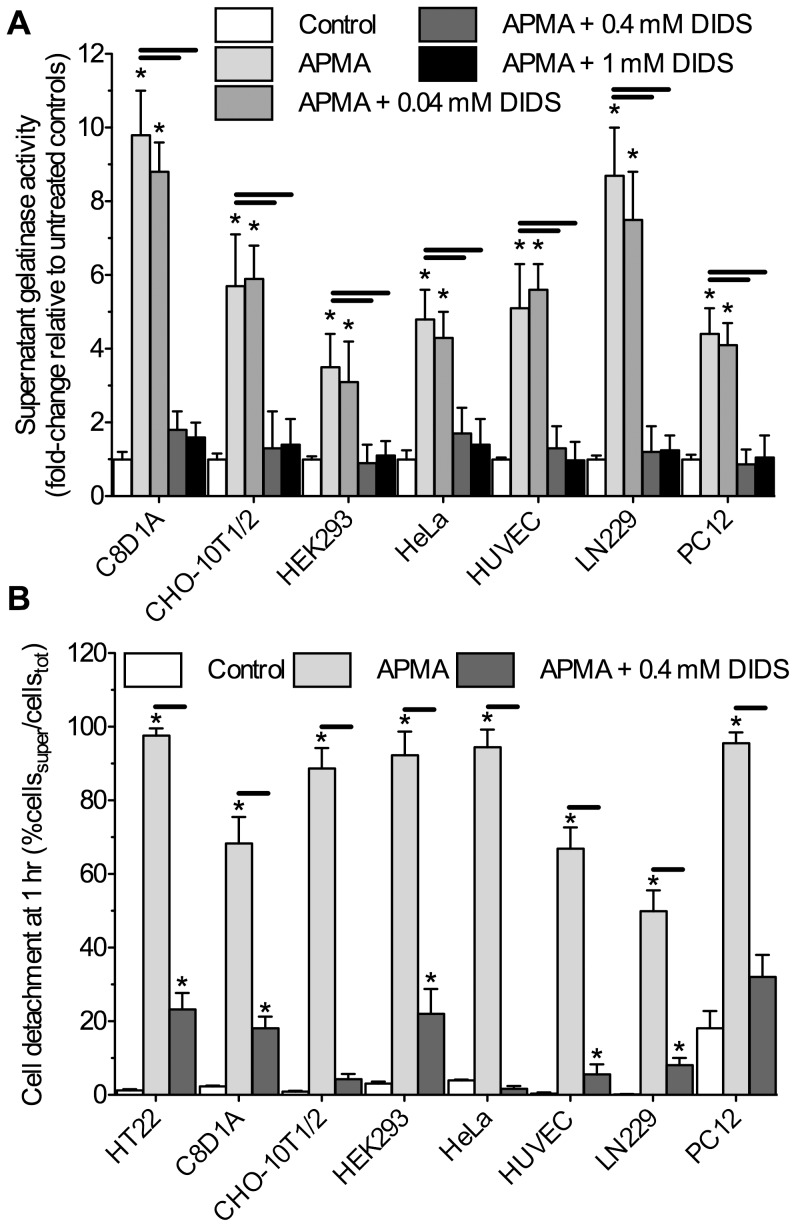
DIDS prevents APMA-induced cellular detachment and extracellular gelatinolytic activity in various cell types in a dose-dependent fashion. (**A**) Summary of the effect of DIDS on APMA-mediated supernatant gelatinolytic activity from 7 additional cell lines treated with 100 µM APMA for 1-hr and analyzed as per Fig. 5 caption. (B) Summary of the effect of DIDS on APMA-mediated cellular detachment from the matrix following 1-hr treatment and analyzed as per Fig. 1 caption. Data are mean ±SEM and experiments were repeated 3 times for each cell type. Asterisks (*) indicate significant difference from normoxic controls; black bars indicate significance between connected treatments (*p*<0.05). Treatments as per Fig. 5 caption.

To test the universality of this inhibition, we also examined the ability of DIDS to impair gelatinolytic activity in a variety of commonly studied cell lines, including rodent type-I astrocytes (C8D1A), glioma (LN229), and mesenchymal cells (CHO-10T1/2), human endothelial (HUVEC), carcinoma (HeLa), and kidney (HEK293) cells, and neuronally differentiated PC12 cells. DIDS had a similar effect in these cell lines as in neurons, and following 1 hour of treatment, reduced APMA-mediated ECF gelatinolytic activity from all cell lines examined in a dose-dependent fashion (*n* = 3 each, Fig. 7A). Furthermore, DIDS prevented APMA-mediated cellular detachment in all cell lines examined (*n* = 3 each, Fig. 7B).

### DIDS inhibits von Willebrand Factor secretion

**Figure 8:**
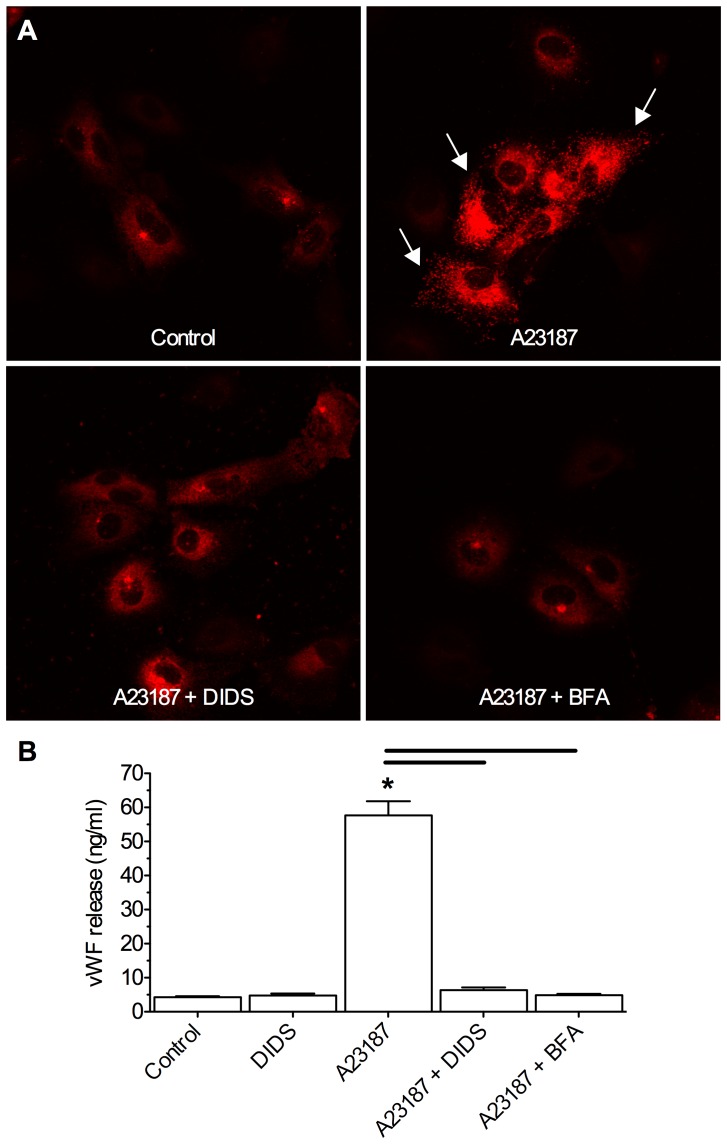
DIDS inhibits stimulus induced vWF release from normoxic HUVECs. The Ca^2+^ ionophore A23187 induces vWF extrusion from HUVECs in a non-pathological model of vesicular release. DIDS abolishes stimulus-evoked vWF release. (**A**) Confocal Z-stack projection fluorescent images of vWF localization (red) in HUVECs treated as indicated. Arrows indicate vWF released extracellularly. (**B**) Summary of supernatant vWF expression measured by ELISA. Data are mean ±SEM from 3 separate 15-min experiments. Asterisks (*) indicate significant difference from normoxic controls; black bars indicate significance between connected treatments (*p*<0.05). Treatments as per Fig. 1 caption, and 10 µl A23187, 1 µg/ml brefeldin A (BFA).

Our observation that DIDS impairs stress- and also APMA-induced MMP-2 and -9 expression and activity in the supernatant, but not the actual production of MMP-2 and -9 in neurons themselves, suggests that DIDS interferes with the efflux of MMPs from cells, and thereby their subsequent activation and enzymatic digestion of neuronal membranes. Since MMP efflux occurs via vesicular release, we hypothesized that DIDS interferes with this mechanism. To test this putative action in the absence of potential proteinase involvement, we utilized a well-established model of vesicular release: that of von Willebrand Factor (vWF) from HUVECs following stimulation with the Ca^2+^-ionophore A23187 [31]. Fifteen minutes of treatment with A23187 induced an ∼10-fold increase in the release of vWF from HUVECs in IHC and ELISA analysis (*n* = 3, Fig. 8A–B). Furthermore, when DIDS was co-applied with A23187, vWF release to the ECF was abolished, while DIDS-treatment alone had no effect on vWF activity. In separate control experiments we co-treated cells with A23187 and brefeldin A (BFA), an inhibitor of vWF release upstream of vesicular function. In these experiments BFA entirely blocked the ability of A23187 to induce vWF release from HUVECs.

## Discussion

We demonstrate that DIDS prevents stress-induced vesicular release of MMPs and subsequent deleterious cleavage of nearby neuronal membranes and cellular detachment from the matrix. Normally, MMP-2 is constitutively expressed at low levels and acts locally to remodel the extracellular matrix during routine maintenance. When neurons are challenged with IS or STS, MMP-2 and -9 protein expression increases as part of the inflammatory response and these pro-enzymes are extruded via vesicular exocytosis to the ECF; where they are activated, and then act locally to digest plasma membranes and induce further MMP production via a feed-forward mechanism [12], [13], [20], [21]. We suggest that DIDS interferes with normal MMP efflux across the plasma membrane and does not directly interfere with proteinase activity, since (1) DIDS markedly reduces exocytosis-dependent MMP protein expression and gelatinolytic activity in the ECF, but not in neurons, (2) a vesicular V-ATPase antagonist similarly impairs MMP protein expression in the ECF and abolishes ECF gelatinolytic activity, and (3) DIDS prevents stimulus-evoked vWF efflux from HUVECs, a well-characterized non-pathological model of vesicular exocytosis that does not involve proteinase activity [31]. Furthermore, this inhibitory action of DIDS on extracellular MMP expression and gelatinolytic activity does not appear to be specific to ischemic pathologies as it occurs during both IS- and apoptotic stress-induced MMP activation and also following direct MMP stimulation with the general proteinase agonist APMA.

Through this mechanism DIDS prevents the extrusion of MMPs into the ECF and reduces resultant deleterious plasma membrane degradation. DIDS has previously been suggested to be cytoprotective against pathological insults. Most profoundly, DIDS has shown promise against ischemic pathology in heart and brain, ameliorating up to 90% of cell death [30], [35], [36], [37]. In addition to ischemia there is evidence that DIDS is protective against other pathologies, including: beta-amyloid formation in cortical neurons; arsenic-, STS-, and ethanol-induced apoptosis in leukemia cells, cortical neurons, and cardiomyocytes, respectively; and volume-dependent apoptosis in cardiomyocytes, epithelial cells, and neurons [38], [39], [40], [41]. Protective effects of DIDS are usually attributed to blockade of anion channels or anion exchangers and associated reductions of Cl^−^ or reactive oxygen species flux that regulate cell volume or downstream stress pathways mediated by toll-like receptors, mitogen-activated protein kinases, protein kinase C, or phosphatidylinositol 3-kinase/Akt [30], [35], [36], [39], [42], [43]. Our results suggest a novel mechanism through which DIDS prevents stress pathway activation and plasma membrane degradation by inhibiting vesicular-dependant proteinase extrusion. MMPs are critical to programmed plasma membrane digestion and also cleavage-mediated activation of extruded inflammatory signals during pathology. Therefore, inhibition of MMP release from dying cells at the rim of the infarct core would prevent the extracellular translocation and activation of pro-apoptotic and inflammatory mediators and may retard penumbral spread during ischemic stress.

Notably, the ability of DIDS to impair proteinase-mediated gelatinolytic activity and membrane cleavage by preventing MMP release may be ubiquitous among cell lines derived from various tissues and organisms since DIDS potently inhibited gelatinolytic activity and proteinase-mediated cellular detachment in the eight murine and human-derived cell lines examined in this study. Furthermore, DIDS has been shown to preserve membrane integrity in primary hippocampal and cortical slice cultures *in vitro* [28], [29], and it is therefore tempting to speculate that membrane preservation in these primary cell and tissue models was similarly mediated by inhibition of proteinase activity by DIDS. Interestingly, DIDS has also been reported to inhibit synaptic trafficking of glutamate and ATP in mammal brain slices and synaptosomes, both of which are vesicular-dependent processes [44], [45]. These results indicate that the inhibitory action of DIDS on vesicular release is conserved in primary cells and tissues. Further experiments are warranted to examine this putative mechanism of DIDS *in vivo*, and particularly to determine whether DIDS-treatment affects ischemia-induced infarct and penumbral expansion, or blood brain barrier degradation in intact brain.

One component of physiological MMP function and regulation that we do not explore in the present study is the effect of pathological treatments ±DIDS on the transcriptional regulation of MMPs. Others have demonstrated that mmp2 and mmp9 mRNA are upregulated by ischemic stress *in vivo* [20], [46], [47], [48], [49]. In general, MMP-2 transcriptional activation is rapid and occurs in a time window of 2–12 hrs following stress, while MMP-9 mRNA transcription is delayed until 4–8 hrs following insult and persists for 24–48 hrs. Thus at the time point at which we examine stress-mediated changes in MMP protein expression in the present study (6 hrs), we expect transcriptional activation of both mmp2 and mmp9 to be initiated. Although we do not measure MMP-related transcriptional changes directly in the present study, our data demonstrating an increase in the expression of latent (i.e. transcriptionally regulated [15]) MMP-2 and -9 isoforms following IS or STS treatment indirectly demonstrate that transcription events have been activated. Furthermore, our failure to detect latent MMP-9 proteins in control cells also suggests that the stress-induced increase in the intracellular expression of this isoform is mediated by transcriptional activation. Since DIDS has no effect on stress-induced changes to the intracellular expression of proMMP-2 or -9 protein isoforms in any treatment, it is unlikely that DIDS effects the transcriptional regulation of MMPs.

Another limitation of our study is that the Western blot and zymography assays employed induce the dissociation of MMPs from TIMPs, and therefore these results may not directly reflect changes of MMP activity *in situ*. Furthermore, like MMPs, TIMPs are also secreted to the ECF via vesicular exocytosis [15]. Others have reported that DIDS impairs vesicular exocytosis of ATP and glutamate [44], [45], and together with our present observations that DIDS similarly impairs exocytosis-mediated MMP and vWF secretion, these data suggest that this effect of DIDS on vesicular exocytosis is not specific to MMP extrusion. Therefore, it is likely that DIDS similarly impairs TIMP protein secretion and expression in the ECF. However, reduced extracellular TIMP protein expression would increase extracellular MMP activity [15], which would in turn increase the expression of the active form of MMPs in the ECF and exacerbate pathological membrane degradation. Instead we observe minimal expression of either the latent or active MMP-2 and -9 isoforms in the ECF of DIDS-treated neurons, and membrane degradation is abolished. Therefore we conclude that *in vitro*, either MMP secretion alone is inhibited, or more likely that both MMP and TIMP secretion is inhibited, but to an equal degree or at least in favor MMP inhibition (i.e. TIMP protein expression≥MMP protein expression). Nonetheless, TIMPs are also secreted by nearby stromal cells *in vivo*. Therefore, although DIDS reduces MMP (and presumably TIMP) secretion from neurons, concomitant inhibition of TIMP release from non-neuronal cells may actually augment enzymatic activity *in vivo*.

In summary, we present evidence that DIDS inhibits the stress-induced extracellular accumulation and digestive activity of MMP-2 and -9, which is dependant on vesicular exocytosis. Through this mechanism, DIDS preserves neuronal membrane integrity and cellular adhesion to the matrix during ischemic or apoptotic insults. Indeed, DIDS inhibits or entirely abolishes vesicular-dependant functions in a dose-dependent manner in a variety of pathological and non-pathological models of vesicular activity across several cell types from both human and murine sources. Targeted modulation of vesicular release offers therapeutic potential in pathologies related to malfunctioning vesicle release pathways, particularly ischemic inflammation and spreading death in the penumbra.

## Supporting Information

Video S1 **24-hr video of neurons treated with normal growth media.** HT22 murine hippocampal neurons treated with normal growth media were visualized using DIC imaging on a confocal microscope. Image frames were taken at 5-min intervals for 24-hrs and videos are displayed at 10 frames/sec.
(MOV)

Video S2 **24-hr video of neurons treated with IS.** HT22 murine hippocampal neurons treated with ischemic solution (IS) were visualized using DIC imaging on a confocal microscope. Image frames were taken at 5-min intervals for 24-hrs and videos are displayed at 10 frames/sec.
(MOV)

Video S3 **24-hr video of neurons treated with IS+DIDS.** HT22 murine hippocampal neurons treated with ischemic solution (IS)+400 µM DIDS were visualized using DIC imaging on a confocal microscope. Image frames were taken at 5-min intervals for 24-hrs and videos are displayed at 10 frames/sec.
(MOV)
